# Nature and Nurture of Human Pain

**DOI:** 10.1155/2013/415279

**Published:** 2013-04-02

**Authors:** Inna Belfer

**Affiliations:** Departments of Anesthesiology and Human Genetics, University of Pittsburgh, Pittsburgh, PA 15213, USA

## Abstract

Humans are very different when it comes to pain. Some get painful piercings and tattoos; others can not stand even a flu shot. Interindividual variability is one of the main characteristics of human pain on every level including the processing of nociceptive impulses at the periphery, modification of pain signal in the central nervous system, perception of pain, and response to analgesic strategies. As for many other complex behaviors, the sources of this variability come from both nurture (environment) and nature (genes). Here, I will discuss how these factors contribute to human pain separately and via interplay and how epigenetic mechanisms add to the complexity of their effects.

## 1. Nurture and Pain

Pain perception (meaning not just physiological processing of nociceptive signal but rather conscious recognition and awareness of painful stimulus) can be modulated and modified (enhanced or abolished) by many “environmental” factors including psychological and personality-related factors such as previous pain experiences, emotionality and cognition, somatization and catastrophizing, presence of acute and chronic stressful life events, fatigue, anxiety, fear, boredom and anticipation of more pain, as well as socioeconomic factors (e.g., social support, acceptance, incentives, education, occupation and quality of life). In addition, pain behavior is different among genders and ethnicities, and varies with age. In addition, some clinical and medical factors correlate with risk for increased severity or chronicity of many painful conditions.

### 1.1. Cultural Factors

The experience of pain is one of the fundamental human senses and most ancient protective survival skills. However, the ways in which people express and treat pain change across time and origin and are influenced by cultural and social factors [1–4]. Although there are similarities in word descriptors among cultural groups, with the word “pain” characterizing the most intense and unpleasant discomfort, the word “hurt” characterizing less severe discomfort, and “ache” describing minimal and bearable pain [5], cross-cultural differences in the copying styles and attitudes towards pain medication have been also noticed [6]. It has been suggested that “people in Eastern cultures have higher pain tolerance than those in the West” [7, 8]. This assumption is partly caused by the fact that painful rituals and ceremonials are widely accepted in Africa, India, and Middle East, and they may reflect overall pain behavior as turning inward, private and personal experience, with stoicism to be nursed from early childhood. In contrast, other cultures express pain verbally and nonverbally with nonhiding crying and screaming [9]. Such willingness to verbalize pain may “be due to the belief that pain is bad, need not be endured, and should be quickly eliminated” [7]. Cultural factors involve several aspects such as religious beliefs, customs, and social appraisal. However, the majority of pain studies consider cultural differences in terms of ethnicity, assuming “common ground” within each group. Over the past decades, a systematic research based on the biopsychosocial model of pain [10] revealed greater experimental pain sensitivity among African-Americans compared to non-Hispanic Caucasians [11]. Interestingly, this phenomenon has been reported for pain thresholds as well as pain tolerance levels using heat [12–14] and cold [15] stimulation, and pressure [16] and ischemic [17] controlled stimuli. Similar differences have been reported also for acute and chronic clinical pains. Compared to non-Hispanic Caucasians, Latino and African-Americans have greater acute postoperative pain [18], pain following spinal fusion for scoliosis [19], angina during a treadmill exercise [20], and higher or more severe levels of chronic pain related to acquired immune deficiency syndrome [21], glaucoma [22], osteoarthritis [23], and low back pain [24]. All together these ethnic differences in pain are pretty consistent although the underlying mechanisms are unclear [25]. They may reflect the whole array of “environmental” factors that affect minority populations in general such as disparities in socioeconomic status that may lead to undertreated pain [26], high levels of chronic stress due to unfair treatment and discrimination [27], limited social support [28], “John Henryism” [29], and religious coping that are particularly salient for African-Americans and may have an impact on pain experience [27]. The meaning of pain (e.g., pain as retribution versus pain as something to be mastered) can be influenced by sociocultural factors related to ethnic background [30]. These pain appraisals, in turn, can have a major influence on pain-related emotional responses (e.g., depression, guilt, and anxiety) and behavioral responses (e.g., the decision to seek treatment and adherence to treatment regimens) [25]. Ethnic effects may potentially interact with other important variables, such as gender and age, which are known to influence pain perception. 

### 1.2. Demographic Factors

Gender effects on human pain have several aspects. First of all, research showed that gender of experimenter(s) influences the results of pain assessment in laboratory studies. For example, males reported significantly less cold pressor-evoked pain in front of a female experimenter than a male experimenter, while female subjects tended to report higher pain to the male experimenter [31]. Furthermore, a significant interaction of experimenter gender and subject gender on pain tolerance indicated that subjects tolerated pain longer when they were tested by an experimenter of the opposite sex. Additionally, a significant main effect for experimenter gender showed higher pain intensities for subjects tested by female experimenters [32]. On the other hand, recent study in 11,000 electronic medical records containing at least one disease-associated pain score analyzed differences in disease-specific pain reported by men and women and found significant gender specificity [33]. Apparently, women feel pain more intensely than men across almost all disorders. These findings are in line with previous evidence that females are at greater risk for developing several chronic pain disorders such as fibromyalgia, temporomandibular dysfunction, migraine, rheumatoid arthritis, and irritable bowel syndrome [34], and women exhibit greater sensitivity-to-noxious stimuli in the laboratory compared with men [35, 36], for all types of stimulation and tests (reviewed in [37, 38]). In addition, clinical pain is often reported with higher severity and frequency, longer duration, and present in a greater number of body regions in women than in men [39]. Gender affects also analgesic response so that opioids produce greater analgesic responses in women than men [40]. Finally, gender role influences sensitivity to pain in experimental settings [41]. Mechanisms underlying gender effects include biological factors (e.g., contribution of gonadal hormones [42–44]; endogenous and exogenous modulations of pain [45, 46]) and psychosocial factors (e.g., gender roles [47, 48]) and cognitive/affective variables [37, 49], and they have yet to be fully uncovered.

There is increasing recognition that aging can have a profound effect on pain. Pain sensitivity may diminish in adults of advanced age [50]. Examples are the frequent absence of pain in older patients with painful conditions such as myocardial infarction, peptic ulcer disease, and pneumothorax, but the understanding of why older patients with visceral disease are more likely to present without pain is still rudimentary [51]. Similarly, age affects postsurgical pain, for example, older age is associated with less acute [52] and chronic persistent post mastectomy [53] pain. However, the majority of older adults experience pain on a regular basis [54], the incidence of pain is more than the double once individuals surpass the age of 60, and pain frequency increases with each decade (American Geriatric Society; [55]). Also, the assessment and management of pain is more challenging with increasing age due to cognitive impairment and other barriers [56, 57]. Overall, the effect of aging on pain is complex and depends on pathophysiologic, pharmacokinetic, and pharmacodynamic changes in the elderly as well as psychosocial factors such as years of education and marital and socioeconomic status [58]. 

Life style and habits (e.g., exercising, smoking, and drinking) also contribute to human pain perception. Exercise can be both a treatment and a stimulus of pain, so that too much exercise increases pain, while too little exercise may worsen pain through multiple mechanisms (e.g., pain posturing, deconditioned muscle microtrauma, and neuroendocrine responses [59, 60]). Exercise activates endogenous analgesia (in healthy individuals and clinical patients) via triggering the release of beta-endorphins from the pituitary (peripherally) and the hypothalamus (centrally), which in turn enables analgesic effects by activating *μ*-opioid receptors peripherally and centrally, respectively [61]. Therefore, exercising and movement modification during daily activities are effectively used for various chronic pain disorders, including fibromyalgia [62], chronic neck pain [63], osteoarthritis [64], rheumatoid arthritis, and chronic low back pain [65]. Accordingly, people who exercise sufficiently on regular basis may experience pain differently compared to nonexercising individuals, due to direct analgesic effect and also indirectly through psychological mechanisms (such as mood improvement). 

Cigarette smoking and alcohol drinking also have a complex relationship with pain. On the one hand, both nicotine and alcohol have analgesic properties. In general, nicotine administration via nasal spray or transdermal patches reduces pain sensitivity in both smokers and nonsmokers likely resulting from effects at both central and peripheral nicotine acetylcholine receptors [66]. Also smoking a cigarette decreases awareness of and increases tolerance to some experimental pain stimuli [67, 68]. Likewise, orally administered ethyl alcohol (100%), mixed in a 1 : 1 ratio with tonic water at a dose of 2 mg/kg (the equivalent of two cocktails), produced tolerance to experimentally induced pain comparable to 0.17 mg/kg s.q. morphine (11.6 mg in a 70 kg person) [69]. However, multiple clinical pain studies evidenced that smokers and drinkers are at increased risk of developing back pain and other chronic pain disorders [70, 71]. Furthermore, comparisons between the “cases” (smokers and drinkers) and “controls” (nonsmokers and nondrinkers) with chronic pain disorders have repeatedly demonstrated that “cases” have higher pain intensity scores that have greater impact on occupational and social functions [72, 73] This apparent paradox is not only of considerable scientific interest, but also has a clinical relevance in considering patient's habits in the perioperative period and their management in chronic painful conditions. Moreover, a cross-sectional study of young twins demonstrated that smoking, alcohol consumption, and overweight in adolescence correlate with low back pain, and the followup prospective study revealed that smoking also predicted the risk of future low back pain [74]. Thus, life style modification may have long-term effect on adult pain.

### 1.3. Psychosocial/Biobehavioral Factors

Personality, mood, and sleep patterns profoundly contribute to pain perception and behavior. Particularly, catastrophizing as a personality trait (e.g., an exaggerated “negative mental set” associated with emotional distress, magnification of symptoms of distress, and rumination on its possible causes and consequences and sense of helplessness) can heighten the intensity of pain in both experimental and clinical settings. Paraphrasing Daniel Defoe, one can say: “In pain trouble to be troubled is to have your pain doubled.” This correlation has been observed across multiple pain measures and in diverse conditions and populations, including mixed chronic pain, low back pain, rheumatoid arthritis, aversive diagnostic procedures, surgery, dental procedures, burn dressing changes, whiplash injuries, and survey samples of young adults and asymptomatic individuals participating in experimental pain procedures and varsity athletes (reviewed in [75]). Recent study revealed that catastrophizing alters pain modulation in patients with persistent clinical pain [76]. Similar observations have been made for “bad mood” and somatization (e.g., behavior characterized by recurring multiple clinically significant complaints about different physical symptoms and extensive health care seeking). Indeed, somatization as well as increased, intense, or persistent depression and anxiety are the most important predictors of adverse outcomes of invasive interventions for chronic pain [77], and evidence indicated a correlation between pain intensity and presence of somatization [78]. Depressive mood and perceived stress were also found associated with pain sensitivity and were predictive of 2- to 3-fold increases in risk of first-onset temporomandibular disorder [79]. All these factors tend to be interrelated, and they affect pain independently and also through interplay. Recent study, for example, revealed that anxiety, depression, catastrophizing, and somatization significantly correlate with persistent pain in women after mastectomy [80]. Finally, studies in clinical populations, as well as experimental studies in healthy adults, suggest that pain experience and sleep are bidirectionally connected so that severe or persistent pain may disturb the sleep, while sleep disturbance enhances pain [81]. Moreover, night-to-night changes in sleep affect pain report. Hours of reported sleep on the previous night highly significantly predict the current day's pain frequency, and less hours of sleep correlate with greater next-day pain in general population [82]. Sleep problems may mediate the association between enhanced pain perceptions in chronic pain patients and attenuated conditioned pain modulation (one of the mechanisms of central nervous system pain amplification) as it was showed for temporomandibular joint disorder [83] and rheumatoid arthritis [84]. Alterations in sleep may also mediate the effects of pain catastrophizing on clinical pain [85].

### 1.4. Clinical Factors

Although the “environmental” influence on human pain is multidimensional, the role of different groups of factors is not equally important. Research showed that clinical factors have limited power on pain modulation and/or modification. Factors such as patient's knowledge of the diagnosis, disease-related variables, treatment outcome, operative procedures, or the degree of tissue trauma have only little value predicting the risk for long-term pain severity or chronicity [80, 86]. Additional investigation is needed to reveal how these factors correlate with acute pain perception. This may be of clinical importance for acute pain prevention and management to avoid its transformation into disabling chronic pain such as, for example, persistent postmastectomy pain [87].

As described above, the impact of nurture on human pain is multifactorial, and “environmental” factors may shape the effects of each other. The relationships among these factors merit close attention. Future research will uncover the exact connections between psychological and demographic influences on pain. For instance, it is possible that ethnic differences are more pronounced among females than males (or vice versa). Also, it would be interesting to know whether ethnic differences decline or increase with advancing age. While demographic factors modify the effect of other factors on human pain via interact [88], psychological factors, in turn, intervene with demographics, like in the case of catastrophizing mediating sex differences [89] or sleep mediating pain inhibition and spontaneous pain in women [90].

## 2. Nature and Pain

Since “environmental” factors may explain only a small portion of overall interindividual variability in pain sensitivity and pain perception, molecular genetics of pain has been under extensive investigation for the last decades aiming to identify a “missing piece in the puzzle” [91]. Indeed, many “pain candidate genes” have been established using animal studies and then tested in human pain research. Moreover, some of the identified genetic risk factors contributing to pain phenotypes in humans are maintained across multiple species demonstrating extraordinary conservation of nociception and pain behaviors. 

### 2.1. Conservation by Evolution

Fruit flies (*Drosophila melanogaster*) have been used in genetics studies and neuropharmacology for a long time, and they recently have become a powerful model organism for pharmacological pain research [92]. In response to the touch of a probe heated above 38°C, *Drosophila larvae* produce a stereotypical rolling behavior, unlike the response to an unheated probe. However, *Painless* mutants lack this noxious heat response [93]. In the CO_2_ laser beam assay, *painless* gene essential for nociception in *Drosophila larvae* also influenced heat avoidance behavior in adult *Drosophila*, and, as in mammals, the latency of this behavior is inversely related to stimulus intensity [94]. Using genome-wide mutation screen and neuronal-specific RNAi knock-down (over 14,000 genes) in wild-type and *Painless* adult *Drosophila*, hundreds of genes implicated in heat nociception in the fly have been identified, 35% of which were already suspected to be pain genes, and other had no previous functional annotations [95]. One of them was *straightjacket* gene, a member of the **α*2*δ** family of genes that function as subunits of voltage-gated Ca^2+^ channels and control the function and development of synapses [96]. This fly gene has an ortholog gene in mammals, **α*2*δ*3* (33% identical and 60% similar, and the domain structures are conserved throughout evolution [97]. **α*2*δ*3* mutant mice generated by homologous recombination showed impaired acute heat pain behavior, while other behavioral assays were not affected [95]. Most interestingly, fly's *straightjacket* and mammals' **α*2*δ*3* ortholog gene in humans, **α*2*δ*3 (CACNA2D3)*, as predicted, affects heat pain variance in humans: the minor allele of the single nucleotide polymorphism (SNP; the most abundant and common type of genetic variation and markers of choice in human genetic studies) rs6777055 contributed to reduced thermal pain sensitivity in healthy volunteers (i.e., heat wind-up pain model that measures successive increases in perceived pain intensity to a repeated noxious heat stimulus [95]). In addition, the minor alleles of two *CACNA2D3* SNPs (rs6777055 mentioned above and rs1851048) were associated with less chronic pain in sciatica patients “confirming” the reported implication of thermosensitive neurons in human chronic pain. These data reinforce the extraordinary conservation of the neurobiological mechanisms of nociception from its manifestation as avoidance of damage in primitive creatures like flies to the complex sensation of pain in humans. Other genetic factors and molecular pathways related to pain may also be evolutionary conserved, and data from lower species may be easily translated to humans. By cross-referencing fly and mammal data with human genetic information from the public domain-like gene expression profiling or genetic association studies new potential therapeutic targets can be pinpointed [98]. **α*2*δ*3* is a perfect example of such an approach, since it is a close homolog of **α*2*δ*1* which is the molecular target of gabapentin and pregabalin [99], widely used analgesics for neuropathic pain in humans [100]. It is, therefore, possible that novel drug acting via **α*2*δ*3* will appear as powerful analgesic. Pain genetic research in other model organisms such as fish [101] or invertebrates [102] can provide more valuable data on molecular pathways conserved by nature that are involved in human pain and analgesia.

### 2.2. Heritability

Genetic linkage and association studies of human pain are based on the heritability estimates for experimental pain phenotypes and clinical painful conditions that apparently are similar to what has been found in animal models [103]. The heritability of a trait measures the extent to which differences among members of a population can be explained by differences in their genetics [104]. The idea of using twins to measure the influence of heredity dates back to 1875, when the English scientist Francis Galton first suggested the approach (and invented the phrase “nature and nurture”) [105]. Twins offer a precious opportunity to untangle the influence of genes. Investigators may compare the likelihood that identical (monozygotic) twins share a given trait with the likelihood that fraternal (dizygotic) twins share the same trait and quantify the extent to which the genetic variation contributes to the difference. Heritability of many pain phenotypes has been estimated using twin studies; however, those estimates have been calculated for a given population and may vary as function of demographic and other “environmental” effects. Where two correlated phenotypes are examined together, twin studies can estimate the degree to which the phenotypic correlation is mediated by common genetic factors suggesting distinct or overlapping genetic background [104]. Studies show that genetic contributions to, for example, cold and heat pains had little overlap. 60% of the variance in cold-induced pain and 26% of the variance in heat-induced pain were heritable [106] demonstrating the power of thermal sensory testing to distinguish genetic mechanisms of pain processing caused by each type of stimuli. Overall, for experimental pain perception, heritability ranged from 10% (for mechanical pressure thresholds measured via algometry) to 55% (for pinprick hyperalgesia measured via weighted probes) and 61% (for cold pressor test) [38]. Similar findings have been reported for clinical pain, with heritability around 50% for migraine, tension-type headache, and chronic widespread pain, around 35% for back and neck pain, and around 25% for irritable bowel syndrome [107]. High heritability of pain phenotype can either result from large contributions of one or several “major genes,” or small contributions of many [108]. Genetic studies attempted to evaluate both possibilities, with the hypothesis of “rare mutation, rare disease; common mutation, common disease.” Although increasing evidence shows that relatively rare mutations may also cause common and complex diseases like cancer or schizophrenia, former approach seems to work well for studying monogenic pain disorders and polygenic painful diseases, for example, in case of *SCN9A* gene encoding the *α*-subunit of the voltage-gated sodium channel Nav1.7 [109]. 

### 2.3. Monogenic Pain Disorders

The gene *SCN9A* is responsible for three rare human pain disorders. Nonsense mutations cause a complete absence of pain [110], whereas activating mutations cause severe episodic pain in paroxysmal extreme pain disorder and primary erythermalgia [111, 112]. 

Primary erythromelalgia (PE, also called erythermalgia or inherited erythromelalgia (IEM)) is an autosomal dominant disorder with symptoms typically including episodes of burning pain triggered by heat or exercise, together with erythema and mild swelling primarily in the hands and feet [113]. The onset of PE symptoms may occur within the first decade of life, and both the frequency and severity of pain episodes increase with age, with each episode lasting minutes to hours. PE patients do not report autonomic abnormalities (e.g., orthostatic hypotension or gastrointestinal symptoms). To date, a total of fourteen *SCN9A* mutations have been linked to PE [114]. All mutations are in highly conserved residues and have been characterized as gain-of-function mutations [115]. Penetrance for these established mutations appears to be complete; however, de novo mutations (e.g., I848T, and A863P) have been also reported [116]. Interestingly, the A1632E mutation causes both PE and paroxysmal extreme pain disorder (PEPD), also known as familial rectal pain [117]. Symptoms are early onset (developing immediately after birth) and are characterized by life-long pain episodes associated with tonic posturing followed by flushing of the lower limbs that can be either unilateral or bilateral. Nine *SCN9A* mutations have been linked to PEPD [118], most of which have an autosomal dominant pattern of inheritance with complete penetrance, although de novo mutations have been also reported [119]. 

Unlike PE and PEPD, congenital insensitivity to pain (CIP) is autosomal recessive and extremely rare disorder. Although CIP patients exhibit varying degrees of deficits in terms of sensing pain and their response to painful stimuli (e.g., burn, bone fracture, finger and toe mutilation, and visceral pain), other sensory (nonnociceptive) modalities remain intact. To date, thirteen rare *SCN9A* alleles causing CIP have been reported, all leading to loss-of-function mutations occurring in coding regions of the gene [118, 120]. These findings from Mendelian heritable pain disorders illuminating the important role of *SCN9A* variation for human pain led to the hypothesis that common mutations (e.g., SNPs) in the same gene may underlie the differing pain perception in general population. Indeed, from 27 common SNPs genotyped, a significant association was found between pain score and SNP rs6746030 in five tested cohorts; so that the rarer A allele was associated with increased pain scores compared to the commoner G allele in patients with lumbar discectomy, osteoarthritis, sciatica, and phantom pains [109]. The A allele of rs6746030 was also associated with an altered evoked pain thresholds in healthy female volunteers, and the effect mediated through C-fiber activation [109]. These data supported the theory that rare mutations cause rare disorders, while common mutations contribute to common phenotypes, and further studying of rare monogenic pain conditions may provide evidence for the role of pinpointed genes for “general” pain phenotypes. 

Another group of such disorders consists of five types of hereditary sensory neuropathies (HSNs) characterized by sensory loss, insensitivity to pain, and a variable degree of muscle weakness and wasting, as well as autonomic features [121]. Molecular genetic studies have assigned a distinct genetic loci for each type: HSN I to chromosome 9q22.1–22.3 and missense mutations in *SPTLC1* [122]; HSN II to chromosome 12p13.33 and *HSN2*, an alternatively spliced exon of *WNK1* gene [123]; HSN III to chromosome 9q31 and *IKBKAP* mutations [124]; HSN IV to chromosome 1q21-q22 and *NTRK1* mutations [125]; and HSN V to chromosome 1p13.1 and *NGFB* [126, 127]. However, similarly to *SCN9A* alleles, these HSN genes may influence more than one type of rare pathological pain conditions; a novel mutation in *NTRK1* gene known to be responsible for HSN IV apparently causes HSN V [128], and other *NTRK1* mutations and polymorphisms result in CIPA (congenital insensitivity to pain with anhidrosis) [129]. 

The final group of pain disorders with a very prominent genetic impact is three types of familial hemiplegic migraine (FHN), a rare monogenic dominant autosomal disease due to mutations in a single gene [130]. Mutations on *CACNA1A* on chromosome 19p13 (FHM1), *ATP1A2* on chromosome 1q23 (FHM2), and *SCN1A* on chromosome 2q24 (FHM3) were identified as causing FHM [131]. Besides the classical phenotype, alleles in each gene may be associated with a broader spectrum of clinical features, making each FHM type a complex channelopathy [132]. Future research may reveal overlapping genetic background among FHM types similar to other rare pain disorders. 

Mendelian heritable pain disorders have provided insights into general human pain mechanisms highlighting genes encoding for sodium and calcium channels and suggesting new analgesic drug targets that may be of relevance for the treatment of both rare and common pain diseases [133]. It is attractive to propose that polymorphisms in these gene loci that produce quantitative rather than qualitative changes in gene function may underlie less severe but more frequent human pain conditions. Knockout of a pain gene is very rare in nature. Because pain mechanism genes are too well conserved to explain individual variation, research attention has shifted to studying pain susceptibility genes underlying common painful diseases.

### 2.4. Polygenic Painful Diseases

Pain perception in general population is one of the most complicated measurable traits and a complex genetic trait of polygenic nature [134]. “Common” painful diseases are much more heterogeneous and multifactorial compared to Mendelian disorders, since they are induced and influenced by both diverse environmental factors (e.g., trauma, surgery, or disease) and a complex array of various genetic factors [135]. Although linkage analysis studies of Mendelian pain disorders have pointed to genes with rare as well as common variation that has significant role in complex pain disorders, many more genetic variants are expected to contribute. In fact, during the past fifteen years candidate gene association studies have proved to be a useful tool in revealing influence of many genes on different types of human pain, and the list of “pain genes” is rapidly increasing [136]. Since there is a high comorbidity between clinical pain conditions, it is expected that the identified genes will be implicated in more than one condition, and findings from recent studies support this assumption. A common *SCN9A* allele discussed above contributed to pain in four frequent chronic painful diseases suggesting that they may share the same genetically determined pain pathway [109]. A common haplotype in *GCH1* gene, encoding for the rate-limiting enzyme for the synthesis of tetrahydrobiopterin (BH4), an essential cofactor for catecholamine, serotonin, and nitric oxide productions, was protective for persistent sciatica [137], advanced cancer pain [138], and a pain-related treatment outcomes in disk degenerative disease patients [139] suggesting its overall ability to suppress chronic pain. A common amino acid-changing allele in *KCNS1* gene, encoding for potassium channel alpha subunit involved in neuronal excitability, also affects multiple chronic pain conditions, so that the “valine risk allele,” was significantly associated with higher pain scores in five of six independent patient cohorts assayed [140]. The universal feature in all patient populations tested in the studies of these three genes was well-defined “organic abnormality” causing chronic pain. In contrast, the group of chronic “functional” or “idiopathic” pain syndromes is more challenging for assessment due to the lack of obvious origin of pain(s). This group consists of temporomandibular joint disorders (TMJDs), fibromyalgia syndrome (FMS), irritable bowel syndrome (IBS), chronic headaches, interstitial cystitis, chronic pelvic pain, chronic tinnitus, whiplash-associated disorders, and vulvar vestibulitis (VVS). Although the exact pathophysiological mechanisms that underlie the majority of these conditions are poorly understood, several shared genetic factors have been reported which mediate pain amplification and underlie substantial individual variability in related pain phenotypes [135]. Common polymorphisms in the promoter region of the serotonin transporter gene (*SLC6A4*) are associated with fibromyalgia [141], chronic fatigue syndrome [142], migraine headache [143], and TMJD [144]. *COMT* gene is a major “pain gene” contributing to a variety of pain phenotypes and particularly to “idiopathic” pain conditions. Enzyme encoded by this gene (COMT) metabolizes catechol neurotransmitters dopamine, noradrenaline, and adrenaline that are involved in various physiological functions closely related to pain [145]. Functional *COMT* variants include at least two SNPs and common haplotypes (sets of coexpressed SNP alleles): the most studied SNP rs4680, also known as Val158Met, leading to a three- to four-fold reduced activity of the COMT enzyme [146]; SNP rs2097603 in the *COMT* P2 promoter region also affecting enzyme activity [147]; and three major haplotypes formed by four SNPs (one located in the *COMT* promoter region (A/G; rs6269) and three in the *COMT* coding region at codons his62his (C/T; rs4633), leu136leu (C/G; rs4818), and Val158met (A/G; rs4680)) leading to the largest, up to 20-fold difference in enzyme activity, via changes in the stability of the secondary mRNA structure [148]. It was proposed that individuals with *COMT* alleles resulting in high enzyme activity will metabolize catecholamine more efficiently and presumably have reduced catecholamine-mediated neurotransmission [149]. With respect to “idiopathic” pain, Israeli FMS patients who were homozygotes for the *COMT* Met158 allele showed increased sensitivity to pain and the number of pressure points compared to other genotypes [150]; Brazilian FMS patients carrying minor alleles of two SNPs, rs4680 and rs4818, reported more pain [151], and Spanish FMS patients carrying minor allele for SNP rs6269 demonstrated more severe symptoms [152]. *COMT* Val158Met SNP showed differential allelic distribution among patients with nonfamilial migraine and healthy controls, so that Met allele was over represented in the migrainous patients [153]. In a large population-based study, a lower prevalence of headaches was found in Val158 homozygotes [154], while the Met158 allele was significantly associated with higher pain intensity of headache (in another cohort) [155]. *COMT* functional haplotypes correlate with the risk of developing myogenous TMJD [156]. Homozygosity for *COMT* Met158 allele predicts strongest placebo effect in IBS patients [157], while the IBS Val/Val carriers exhibited significantly increased bowel frequency and other IBS-specific symptoms [158]. Finally, in the study of acute whiplash-associated pain, homozygotes for *COMT* pain vulnerable haplotype were more likely to report moderate-to-severe neck pain after motor vehicle collision and also longer time to physical and emotional recovery, indicating that *COMT* genetic variation affected both somatic and psychological responses in the allele carriers [159]. Therefore, it seems like all those “functional” pains have a common catecholamine-related underlying pathway influenced by *COMT* variants. Other genes may affect these disorders through other mechanisms, such as recent findings of *SCN9A *allele described above influencing interstitial cystitis/bladder pain syndrome [160]; functional alleles in interleukin-1 receptor antagonist (*IL1RN*) and melanocortin-1 receptor (*MC1R*) genes increasing the risk of VVS [161]; or proinflammatory cytokine gene polymorphisms in *IL-6* and *TNF-*α** changing individual susceptibility to IBS [162]. On the other hand, *COMT* effects are not limited to “idiopathic” pain disorders and have been found in other pain models. *COMT* Val158Met and haplotypes showed significant associations with the maximum postoperative pain rating (acute clinical pain responses) in third molar extraction model [163]. In addition, *COMT* haplotype was associated with less chronic postoperative pain (greater improvement in Oswestry Disability Index scores) in patients with disk degenerative disease one year after surgery, suggesting that *COMT* variation may be predictive in terms of treatment outcome [164].

Genetic studies of certain pain conditions may be more challenging than others due to higher degree of phenotypic diversity. For example, acute and chronic low back pain (LBP) may be influenced by different genetic factors, depending on underlying primary causing pathology such as lumbar disc degeneration or other condition affecting the spine (e.g., herniated disc, spinal stenosis, osteoarthritis, spondylolysis, fractures, deformities, ankylosing spondylitis, bacterial infection, or tumor), irradiating pain from hip diseases, spine injury or just overuse of muscles, ligaments, and joints. Such a heterogeneous etiology and pathophysiology require careful consideration of all significant clinical factors as covariates in association analysis and meta-analysis of LBP genetic data [165]. Nevertheless, studies investigating the genetic mechanisms of LBP related to lumbar disc disease found two alleles of collagen IX gene associated with sciatica and lumbar disc herniations and an aggrecan gene polymorphism, a vitamin D receptor, and matrix metalloproteinase-3 gene alleles contributing to pain outcomes in disc degeneration patients [166]. Another condition with high heterogeneity of pain phenotypes is sickle cell disease (SCD), one of the most common inherited diseases worldwide that is characterized by chronic pain syndromes, acute recurrent painful crises, neuropathic pain, or a mixture of all three [167]. The arrays of SCD pains include extremely diverse phenotypes, from infection-related inflammatory pain to priapism; however, shared genetic background may underlie individual variability across multiple SCD pain phenotypes. To date, only one of them, acute painful crisis, has been studied genetically. Common SNPs in fetal hemoglobin gene were associated with pain crisis rate in SCD patients [168]; polymorphisms in human platelet alloantigen family genes contribute to pain crisis risk [169], and vascular endothelial growth factor gene variants were correlated with pain crisis type and duration [170]. 

### 2.5. Evoked (Experimental) Pain

Many heterozygous clinical pain phenotypes can be dissected for genetic analysis using “intermediate” pain phenotypes or “endophenotypes”—such as cytokine profiling, brain imaging, or quantitative sensory testing (QST) variables—measurable components along the pathway between the complex trait and genotype. Endophenotypes represent simpler clues to genetic underpinnings than the syndrome itself, promoting the view that such a syndrome can be decomposed or deconstructed, which can result in more straightforward and successful genetic analysis [171]. Characteristics of the endophenotype include that it should affect a given complex disorder, vary continuously in the general population, can be measured across several levels of analysis, be associated with causes rather than effects of disorders, and, most importantly, be heritable [172]. QST variables, assessing somatosensory evoked responses to noxious or innocuous stimuli using controlled mechanical, chemical, electrical, and thermal test modalities [173], have been increasingly shown to be predictive or correlate with various clinical pain conditions such as osteoarthritis [174] or neuropathic pain syndromes [175]. Each QST modality evaluates different mechanisms of pain processing, and QST is widely used in pain research to detect perception threshold values regarding touch (A beta fibers), warmth (C fibers) and cold (A delta fibers), and heat pain (C fibers) [176]. Studies in healthy volunteers and samples of patients show that variability across QST phenotypes is genetically determined. In pain-free subjects, *COMT* functional haplotypes contribute to variability in thermal and other evoked pain sensitivity [177]. Individuals with low-expressing alleles in the serotonin transporter gene (*5HTT or SLC6A4*) exhibit significantly reduced conditioned pain inhibition for pressure pain thresholds and heat pain [178]. A functional genetic polymorphism in the vanilloid receptor ionophore gene (*VR1*) moderates heat/capsaicin sensitivity in healthy humans [179]. Increased sensitivity-to-thermal pain has been observed for redheads with an aberrant melanocortin-1 receptor gene (*MC1R*) [180]. In clinical settings, only a couple of studies investigated intermediate phenotypes (in addition to clinical pain symptoms) in relation to genetic influences. FM patients with *COMT* Met/Met genotype (Val158Met SNP) showed higher sensitivity to thermal and pressure pain stimuli than patients carrying the Val alleles [181]. A study within the German Research Network on Neuropathic Pain showed the transient receptor potential channel gene polymorphisms contributing significantly to the somatosensory abnormalities of neuropathic pain patients [182]. Specifically, transient receptor potential ankyrin 1 710G > A (rs920829, E179K) was associated with the presence of paradoxical heat sensation, and transient receptor potential vanilloid 1 1911A > G (rs8065080, I585V) was associated with cold hypoalgesia. These findings are very promising and lead to more in-depth examination of genetic background of QST-produced endophenotypes and their value for studying the genetic effects on complex pain conditions like chronic neuropathic pain. 

### 2.6. Analgesic Response

Investigation of the nature of analgesia and analgesic responses is a new but rapidly developing field attracting increasing attention in light of recent studies of pharmacogenetics and pharmacogenomics of pain. Pharmacogenetics describes the effects of polymorphic genes on the enzymes that metabolize drugs, and the genetic differences in metabolic pathways can affect individual responses to drugs, both in terms of therapeutic effect as well as adverse effects [183]. Thus, pharmacogenetics refers to the study of inherited differences (e.g., effects of genetic polymorphisms) in drug metabolism and response. Pharmacogenomics, on the other hand, refers to the general study of all the many different genes that determine drug behavior. Both approaches have to consider two different genetic substrates to determine the outcome of pharmacotherapy. The first is the genetic contribution of a variety of different pain types, and the second is the genetic influence on drug effectiveness and safety. Both approaches aim to explore the ways the genetic effects can be used to predict whether a patient will have a good response to a drug (e.g., pain relief), a bad response to a drug (e.g., worse pain and/or side effects), or no response at all. This is particularly important for clinical pain management since many painful conditions are resistant to pain killers, and most pain drugs have serious adverse effects. Many genes from the “human pain genome” may influence analgesic response, much like they do for other pain behaviors including human nociception and pain perception. Functional mutations in these genes modulate a number of pain drug parameters including absorption, metabolism, receptor availability, and secondary messenger signaling [184]. 

The most ancient and powerful analgesics are opiates, although they have very different effects on individuals, from pain relief to secondary hyperalgesia, with a wide range of side-effects including sedation, respiratory depression, constipation, and addiction. Recently, several studies found that genetic polymorphisms greatly affect both analgesic effects of morphine and other opioid drugs and their adverse effects. Since the mu-opioid receptor is the primary site of action for both exogenous (e.g., morphine) and endogenous (e.g., enkephalins and endorphins) opioids, its encoding gene, *OPRM1*, has been the primary candidate in pharmacogenetic and pharmacogenomic studies. It was shown that healthy individuals who are homozygous for the 118G allele (A118G SNP) require 2–4 folds higher opioid consumption to achieve analgesia for evoked pain when compared to individuals not carrying the genotype [185]. Similar findings have been reported in clinical pain sample of patients with acute postoperative pain after total knee arthroplasty showing that 118G homozygotes indeed required more morphine consumption to get sufficient analgesia than those homozygous for the major A118 allele [40]. Interestingly, 118G homozygous carriers also had decreased incidence of opioid-induced nausea and vomiting compared to 118A homozygotes [186]. Another study in female-only sample of acute postoperative pain following hysterectomy or myomectomy revealed the association of 118G allele with higher fentanyl consumption for adequate pain relief but not with postoperative nausea and vomiting induced by fentanyl intravenous analgesia [187]. Finally, a recent study in patients undergoing radical gastrectomy showed the interaction between *OPRM1* A118G SNP and cytochrome P450 3A4 (*CYP*3*A*4)*18B polymorphisms that jointly affect postoperative fentanyl analgesia so that patients with *OPRM1* AA and *CYP*3*A*4*1*18B genotypes received fewer fentanyl doses compared with other genotype groups [188]. These findings are in line with previously reported results on a combined effect of *OPRM1* and *COMT* common functional polymorphisms on morphine postoperative analgesia and central side-effects. The heterozygous patients with *OPRM1* A118G and *COMT* G1947A SNPs consumed significantly less morphine in the postanesthetic recovery room and 48 hours after surgery compared with homozygous patients, and nausea and sedation scores were also significantly lower during all observed postoperative periods for heterozygous patients [189]. In addition to these adverse effects, A118G has been shown to decrease pupil constrictor [190] and respiratory depression [185]. 

*COMT* may contribute directly to morphine efficacy. Cancer patients with the Val/Val genotype (Val158Met SNP) needed more morphine compared to those with the Val/Met or Met/Met genotypes [191]. Other *COMT* SNPs could also play a role in morphine efficacy since carriers for the most frequent haplotype constructed from ten *COMT* SNPs, in addition to the Met allele, need lower morphine doses than patients not carrying that haplotype [192].

Another gene involved in opioid pathway is *ABCB1/MDR1* gene encoding for adenosine triphosphate-binding cassette, subfamily B, member 1, a drug efflux transporter, considered to be a major component of the blood-brain barrier and a major determinant of morphine bioavailability in the central nervous system [193]. Its functional exonic SNP C3435T showed significant association with fewer morphine side-effects in postoperative patients [194]. Moreover, based on the individual's C3435T genotypes and their combined effect with *OPRM1* A80G SNP, Campa et al. were able to predict patients as being “strong responders,” “responder,” or “nonresponders” for morphine pain relief, with sensitivity close to 100% and specificity more than 70% [195]. These gene × gene interactions seem to have the biggest effect on analgesia-related phenotypes. In addition to the aforementioned 3435C > T *ABCB1* alleles, 1236C > T and 2677G > T/A protect against the respiratory depressive effects of fentanyl [196]. 

Besides morphine and fentanyl, codeine also acts predominantly on mu-opiate receptors. Its metabolizing enzyme, cytochrome P450 2D6, is encoded by gene *CYP2D6*. While individuals lacking *CYP2D6* function suffer from poor codeine analgesia, *CYP2D6* duplication genotype carriers (with some individuals inheriting up to 13 copies of the gene, arranged in tandem) may experience exaggerated and even potentially dangerous opioidergic effects due to ultrarapid codeine metabolism [197]. *CYP2D6* genotypes predicting ultrarapid metabolism resulted in about 50% higher plasma concentrations of morphine and its glucuronides compared with the extensive metabolizers. Accordingly, the drug dosage required for the same level of analgesia may differ significantly between the “poor” (two nonfunctional *CYP2D6* alleles), “intermediate” (at least one reduced functional allele), “extensive” (at least one functional allele, e.g., “normal” individuals), and “ultrarapid” (multiple copies of a functional allele and/or an allele, where the mutation confers increased gene transcription) metabolizers. For example, a daily dose of 10–20 mg of nortriptyline may be sufficient for a patient who is a poor metabolizer; however, an “ultrarapid metabolizer” inheriting multiple copies of the gene could require as much as 500 mg a day [198]. While other factors may compensate or modify the effects of these polymorphisms, the determination of patient's *CYP2D6* genotype is clinically relevant for the prediction of analgesic response as well as effects of other CYP2D6-dependent drugs like beta-blockers or antidepressants. 

It is now a well-known fact that red-heads respond differently to local anesthetic drugs or require more of these drugs to get analgesia in clinical settings such as dental office or in response to painful stimuli [180]. The human melanocortin-1 receptor is a key regulator of intracellular signaling to the melanin biosynthetic pathway governing pigment formation, and its dysfunction results in red hear phenotype. Recent studies show that mutations in *MC1R* gene that encodes this receptor and nearly always causes the red hair, also influence opioid analgesic effects. Women with functional *MC1R* SNPs (rs1805007, rs1805008, and rs1805009) displayed significantly greater analgesia from the kappa opioid and pentazocine than all other groups tested [199]. In addition, red-haired volunteers of both sexes showed increased analgesic responsiveness to the mu-opioid selective morphine metabolite morphine-6-glucuronide [199, 200].

Overall, growing evidence suggests that multiple genetic factors influence pharmacokinetics and pharmacodynamics of analgesic drugs, and many common functional polymorphisms may affect analgesic response independently and through interaction [201]. Majority of gene candidates come from genetic studies investigating pain pathways using model organisms that have identified the molecular nature of the transducers and regulatory mechanisms involved in changing neuronal activity, as well as the critical role of immune system cells in driving pain pathways. Mapping these hits in humans using twin and association studies of altered pain behavior revealed the important regulators of the human complex pain system and discovered potential drug targets. In turn, these novel drug targets for pain relief have been validated in transgenic mouse studies [202]. Complementing the genetic studies of pain pathways with traditional neuroscience approaches of electrophysiology and pharmacology provides a perfect insight onto the molecular epidemiology of human pain and creates the “translational research clock” bringing genetics-based discoveries of biological pathways to innovative drug targets and potentially to diagnostic and prognostic markers. 

To date, a relatively limited number of pain genes have been implicated as contributing to human pain phenotypes, while at least 358 genes are thought to be relevant to pain or analgesia according to the Pain Genes Database [203]. About ten genes such as *COMT, OPRM1*, and *TRPV1* have become “gold standard” and the most popular candidates and are constantly tested in different pain models, and studies of functional variants within these genes outnumber studies of all other genes combined [204]. Knowledge of common polymorphisms in other genes with unknown or understudied function is essential for complete picture of the nature of interindividual variability in painful diseases. This knowledge can be generated via the only genotyping method that is not influenced by “gene selection bias”—Genome Wide Association Study (GWAS)—that can highlight gene loci and signaling pathways associated with a given pain phenotype. Certain success using this approach has been done in genetic research of common forms of migraine: four GWASs have successfully identified four new genetic variants including rs1835740 modulating glutamate homeostasis and specifically associated with more severe form (migraine with aura); rs11172113 involving the lipoprotein receptor LRP1; rs10166942 in close proximity to *TRPM8*, which codes for a cold pain sensitivity; and rs2651899 (*PRDM16*) with yet unclear role in migraine [205]. A few pilot GWASs on pain suffered from design flaws such as extremely small sample size [206] or poorly defined pain phenotype in patients with osteoarthritis [207] or endometriosis [208]. Latest GWASs produced more promising results. A GWAS meta-analysis in large samples of chronic widespread pain (CWP) patients found rs13361160 on chromosome 5p15.2 associated with a 30% higher risk of joint-specific CWP and suggested two genes, *CCT5* and *FAM173B*, nearby this locus, as potential targets in the regulation of pain [209]; and a multistage GWAS on opioid sensitivity in healthy subjects showed strong association of rs2952768 and other SNPs within 2q33.3-2q34 locus with increased requirements for postoperative opioid analgesics after painful cosmetic surgery that may further guide investigation of the nature of opioid analgesia [210]. These findings provide valuable rationale for conducting more GWASs in other pain models such as evoked pain or chronic postoperative pain that will lead to better understanding of unique and overlapping genetic mechanisms of pain. To complement the enquiry of the effects of common variants (e.g., SNPs) on pain, an alternative approach is also gaining momentum examining rare genetic mutations that may strongly contribute to the pain conditions in patients with “extreme” phenotypes (such as a very severe or very chronic pain versus no pain after standard procedure or similar injury). Recent state-of-art high-throughput technologies including exome sequencing or whole genome sequencing offer the opportunity to illuminate specific biological pathways associated with a disease, which might lead to new therapies. Unfortunately, human pain genetics has still not taken full advantage of these and other genetic tools and approaches that are widely used in genetic research of other complex traits and behaviors, for example, in psychiatry, for reasons including relatively low funding levels and the relative lack of interested experts in epidemiology, human genetics, bioinformatics, and biostatistics [108]. Current advances in next-generation sequencing, with superfast data turnout, more affordable pricing, and novel analytical applications, give a hope that a large volume of new genetic variants (common mutation with small effects and rare mutations with bigger effects) that may prevent pain by decreasing nociception or increasing analgesia will be identified in the close future. 

## 3. Interplay between Nature and Nurture

Inheritance may play a significant role in human pain, though most of painful disorders are triggered by known or yet unidentified environmental factors. Chronic pain provides the best illustration of the interaction between nature and nurture when it comes to pain. Inflammatory and/or nerve damages are suspected to be the etiology of most chronic pain syndromes like osteoarthritis, diabetic neuropathy, or postherpetic neuralgia, but only a small proportion of those subjected to such injuries actually develop chronic pain, and the degree of pain severity varies a lot across patients. Genes may predispose some to more intense or more chronic pain, but the environment, in turn, can shape the genetic effects. One of the most important environmental factors is stress. Stressful experiences, depending on the type, intensity, and duration of stressor can alter human pain sensitivity by either reducing pain (“stress-induced analgesia”) or exacerbating pain (“stress-induced hyperalgesia”), and the mechanisms of both phenomena are not fully understood. Generally, acute stress induces analgesia (found, e.g., among athletes injured in games and soldiers injured in battle), while the effects of chronic stress in nociception are less predictable [211]. Epidemiological studies have implicated stress (psychosocial and physical) as a trigger of first onset or exacerbation of many painful disorders such as irritable bowel syndrome [212] and low back pain [213]. An individual's response to stress, either physical or emotional, includes activation of the hypothalamic-pituitary-adrenal (HPA) axis, which is accomplished by the secretion of corticotrophin-releasing hormone and arginine vasopressin from the paraventricular nucleus of the hypothalamus [214]. There is evidence that the HPA axis is involved in acute pain and chronic pain. However, it is unclear whether the observed HPA axis abnormalities in stress-related pain syndromes such as fibromyalgia, chronic headaches and temporomandibular disorder reflect preexisting vulnerability to these syndromes or whether chronic somatic symptoms alter HPA axis activity [215]. Thus, HPA-related genes would be obvious candidates to study these complex relationships. Indeed, a recent study investigating the genetic mechanisms of capsaicin-induced pain in healthy subjects found that capsaicin pain levels were influenced by a SNP (rs10877969) within *AVPR1A* gene encoding the vasopressin-1A receptor of arginine vasopressin, but only in male subjects reporting stress at the time of testing [216]. Moreover, the analgesic efficacy of the vasopressin analog, desmopressin, revealed a similar interaction between the drug and acute stress, as desmopressin inhibition of capsaicin pain was seen only in nonstressed men, demonstrating that pain sensitivity and vasopressin analgesia are mediated by a gene-sex-environment interaction. Although this interaction was observed specifically to the chemical/inflammatory modality, most chronic pain states feature inflammation, and the capsaicin test is thought to be an excellent model of human clinical pain [217]. If the genotype and stress (especially chronic stress) modulate the analgesic response also to other drugs, this could have widespread implications for the design of pharmacological studies and pain pharmacotherapy. 

Demographic factors have been reported quite often interplaying with the genetic effects on human pain. For example, increased body mass index mediated the association of a common functional SNP in the gene of brain-derived neurotrophic factor (*BDNF* Val66Met) with fibromyalgia syndrome [218]. Cytochrome P450 gene polymorphisms (*CYP1A1* MspI and HincII genotypes) modified the association between passive smoking and painful dysmenorrhea [219]. Interplay between *β*2-Adrenergic receptor genotype (Gln allele at *ADR *β*2* Gln27Glu), ethnicity and weight contributed to labor maternal pain [220]. Ethnicity and *OPRM*1 A118G genotype were shown as independent and significant contributors to variation in pain perception and postoperative morphine use in patients undergoing cesarean delivery [221]. This genotype interacted with ethnicity also affecting experimental pain sensitivity. The G allele was associated with decreased pain sensitivity among whites only; a trend in the opposite direction emerged in Hispanics [222]. Possible reasons for this dichotomy may involve ethnic differences in gene haplotype structure, or A118G may be a tag-SNP linked to other functional polymorphisms. Common variation in *TRPV1* (Val585 allele) and *OPRD1* (C307 allele) interacted with gender, ethnicity, and temperament contributing to individual variation in thermal pain and cold pain sensitivity in healthy subjects [223]. A study assessing positive affect and pain in fibromyalgia patients revealed a significant gene × experience interaction for *COMT* (Val158Met SNP), such that individuals with met/met genotype experienced a greater decline in positive affect on days when pain was elevated compared to individuals with other genotypes [224]. *COMT* effects on human pain also seem to interact with gender and ethnicity in that the Met allele contributed to variability in short duration cold pain sensitivity (cold pressor test) only in females of Caucasian origin but not in males or other ethnicities [225]. Even more interestingly, the interaction between sex and genotype may lead to opposite effects on pain. A recent study revealed a significant interaction between sex and *OPRM1* A118G genotype regarding the pain intensity in patients with low back pain and sciatica after lumbar disc herniation showing that G allele increases the pain intensity in women but has a protective effect in men the first year after disc herniation [226]. Sex differences have been also reported in heritability of neck pain [227]. These findings demonstrate that understanding the differences in the genetic architecture of complex traits like pain between the two sexes has significant implications for both clinical research and clinical practice, and new analytical algorithms that can detect and quantify the effects of sex on the complexity of quantitative genetic variation are needed. An example of such an approach has been reported recently by Wang et al. who derived a statistical model for mapping DNA sequence variants that contribute to sex-specific differences in allele frequencies, linkage disequilibria, and additive and dominance genetic effects due to haplotype diversity. This model allows a genome-wide search for functional haplotypes and the estimation and test of haplotype by sex interactions and sex-specific heritability [228]. 

Finally, age may also interplay with genetics affecting human pain, especially highly heterogeneous chronic pain disorders such as low back pain [229] or sickle cell pain [230]. Moreover, a study of heritability of neck pain in a large population-based sample of twins showed that despite that genes play a significant role in the liability to this condition (an overall additive genetic component of 44%), the genetic influence becomes gradually less important with the increasing age, and environmental factors dominate almost completely in the older age groups [231]. Thus, the interface between the nature and nurture in relation to human pain may be rather dynamic than static. 

## 4. Epigenetics: Where Nature and Nurture Meet Together

Apparently, nature and nurture are not the only elemental forces at work in regard to pain. There is one other factor in play tied to neither nature nor nurture that in some cases serves as a bridge between the genes and environment and in others operates on its own, shaping individual's pain perception. This third factor, called epigenetics, represents a mechanism by which the environment can directly regulate the translation of the DNA information into proteins via stable and/or heritable changes in gene function that are not intrinsic to the genetic code, but affect gene expression in a tissue-specific manner, resulting in a specific phenotype [232]. Epigenetic mechanisms influence how the genetic code is expressed and how each gene is strengthened or weakened, even turned on or off facilitating the dynamic gene-environment communication. They include covalent modifications of the DNA (methylation) or of the DNA-packaging histones (e.g., deacetylation or phosphorylation) and chromatin remodeling. In addition, regulatory noncoding RNA molecules (e.g., *Xist* and microRNAs) exert epigenetic actions. Epigenome shapes physical structure of the genome: it tightly wraps inactive genes making them unreadable and relaxes active genes making them easily accessible thus adjusting specific genes in response to rapidly changing environment. Some epigenetic processes happen during pregnancy or early life time, other changes appear to occur randomly, and they may be triggered by signals from inside the cell, from neighboring cells, or from environmental factors such as radiation, toxins (chemicals or drugs), nutrition, physical activity, and stress. Importantly, histone modifications and DNA methylation are associated with the subject's age [233]. As with DNA variation, epigenetic modifications may be inherited and may be propagated over multiple cell divisions; however, unlike the DNA sequences, they are flexible enough to respond to modifying influences and can be altered. For example, genes muted by methylation sometimes can be switched back on again. This is very relevant to pain in several aspects. First, epigenetic mechanisms can silence the expression of pro- or antinociceptive genes that contribute to inflammatory and other pain conditions. Next, they may control drug targets and analgesics metabolizing enzymes, thus controlling the pharmacodynamics or pharmacokinetics of analgesics. Epigenetic processes are involved in plasticity and cortical pain processing [234]. Finally, epigenetic techniques such as RNA interference have been employed in pain research to proof the contribution of certain proteins to nociception. It was suggested that future painkillers may work via inhibition of histone deacetylase, to prevent the indirect remodeling of spatial conformation of the chromatin. In addition, it may be possible to suppress pain risk alleles via increased DNA methylation, enhancing microRNAs that degrade mRNA and amplified histone deacetylation. 

 To date, several studies reported promising findings on the role of epigenetics for pain although human data is still very limited. Histone deacetylase (HDAC) inhibitors—compounds that prevent the removal of acetyl groups from histones—can ameliorate pain symptoms in animals and humans via suppression of cytokines [235]. Epigenetic modifications were found to strongly contribute to the development of rheumatoid arthritis by affecting diverse aspects of the disease and modifying gene expression levels and behavior of several cell types, first and foremost joint resident synovial fibroblasts [236]. These cells, when pathologically activated, become apoptosis-resistant, produce chemokines and cytokines thereby promoting inflammation and leading to increased pain and disability [237]. Painful osteoarthritis, the “sister-disease” of rheumatoid arthritis, is also mediated by epigenetic effects [238]. Furthermore, these effects can have an impact on genetic effects in the regulation of gene expression and thus disease susceptibility and severity, and this interplay is bidirectional. Genetic variation can have an effect on methylation of local CpG sites, which may in turn affect gene expression and phenotype [239, 240]. Indeed, an investigation of the epigenetic regulation of the osteoarthritis susceptibility-containing gene *GDF5* demonstrated that DNA methylation can affect the allelic expression imbalance between the C and T alleles of the *GDF5* SNP rs143383, such that loss of methylation of the 5′UTR leads to a significantly greater imbalance in expression between the two alleles influencing their penetrance in susceptibility to osteoarthritis [241]. Similar epigenetic mechanisms are suggested at play in chronic pain conditions shaping the related vulnerability and resilience factors [242, 243]. For example, epigenetic silencing of a gene for the extracellular matrix protein SPARC (secreted protein, acidic, and rich in cysteine), protective against accelerated disk degeneration, is linked to chronic low back pain [244]. Epigenetic changes also influence steroid responsiveness and opioid sensitivity impacting on the efficacy and safety of multiple drugs used in pain management [245], at least in animal models, suggesting future clinical targeting the epigenetic machinery for pain relief [246]. 

 Epigenetic inheritance adds another dimension to the complexity of the nature and nurture effects on human pain continually adjusting individual's gene expression to fit the changing environment without altering DNA code and translating environmental signals to the fixed genome. Epigenetic analysis may identify mechanisms critical to the development and/or persistence of painful diseases and may provide new pathways and target mechanisms for future drug development and individualized pain medicine. Current investigation of genome-wide distribution of acetylation and methylation “tags”—places along the genome where methylation changes the pattern of gene expression—in multiple human cell lines leads to epigenome sequencing, creation of DNA methylation and histone modification maps and evaluation of individual's unique epigenetic profile—an epigenetic signature that determines the level of gene expression, including pain genes. Further, exploring the epigenetic mechanisms of gene regulation in pain and other complex traits and diseases may explain some of the missing heritability identified in GWAS studies [247]. 

In summary, complex traits such as pain are highly heritable, and further intensive scrutiny of DNA sequences themselves may reveal many more important genetic variants with larger or smaller effect size. Modern genetics and genomics provide with powerful tools allowing us to investigate shared biological processes that are common to all individuals (e.g., molecular pathways of nociception) and to dissect the genetic basis for interindividual differences in pain susceptibility, behavior, and pathology [248]. Understanding the genetic basis of these differences is critical to elucidate the molecular basis of pain sensitivity, variable responses to analgesic drugs, and, ultimately, to individualize treatment of pain and improved public health [248]. However, the development of personalized analgesic treatments requires a more complete knowledge of the effects and range of genetic modulation of pain as well as gene-gene and gene-environment interactions in response to analgesics [249]. Well-designed genetic and epigenetic studies of human pain with deep phenotypic data and clear definition of outcomes help to evaluate both genetic and environmental factors influencing human pain perception and how they can be manipulated via epigenetic processes. Clinical genetic methods may illuminate the cause-and-effect relationship between pain and comorbid traits [250]. An integrated genetic and epigenetic approach to pain is in need [251]. With recent advantages in phenotyping and genotyping of human pain [252, 253], new initiatives of the National Institutes of Health including the recently created Roadmap Epigenomics Program [254] and Mapping Consortium [255], and breaking-through technologies and analytical approaches, it will become possible in the near future to develop predictive algorithms based on the unique personal profiling enabling individualized clinical decision making regarding efficacy and risk of pharmacotherapies, behavioral therapies, and invasive procedures. When we completely elucidate the nature and nurture of human pain, we will be able to control it, finally.
